# Delayed cutaneous wound healing in young and old female mice is associated with differential growth factor release but not inflammatory cytokine secretion

**DOI:** 10.1007/s10522-024-10179-7

**Published:** 2025-01-08

**Authors:** Melissa Plum, Justus P. Beier, Tim Ruhl

**Affiliations:** Department of Plastic Surgery, Hand Surgery-Burn Center, University Hospital RWTH Aachen, Pauwelsstraße 30, 52074 Aachen, Germany

**Keywords:** Tissue regeneration, Dermal injury, Inflammation, Immunosenescence

## Abstract

The capacity for tissue repair during wound healing declines with age. A chronic low but systemic inflammatory status, often called “inflammaging”, is considered a key factor that contributes to impaired tissue regeneration. This phenomenon has been substantiated by an increased number of immune cells in wound-tissue of old mice. Although immune cells coordinate an inflammatory response by their secretome the composition of the wound milieu has not been examined. In young (2 months) and old (18 months) female mice, excision wounds were induced using a punch biopsy device, i.e., the healing progress occurred through secondary intention. The closure rate was analyzed for 7 days. At days 1, 3 and 7 post-surgery, wound specimen were investigated for immunohistochemical detection of granulocytes, M1-macrophages and mesenchymal stem cells of the skin. The concentrations of inflammatory cytokines and regenerative growth factors were determined in tissue homogenates by ELISA. The carbonyl assay was used to determine protein oxidation. In old mice, the wound closure was delayed between days 1 and 3 post-surgery, as was the peak of immune cell infiltration. There was no age effect on the concentration of inflammatory cytokines, but wounds of young animals contained higher number of mesenchymal stem cells and increased levels of growth factors. Protein oxidation was increased with age. The present study suggests that a reduced regenerative capacity rather than an enhanced inflammatory score affected the tissue regeneration process in old mice.

## Introduction

Physiological wound healing follows three consecutive phases. The initial is the inflammatory phase that recruits neutrophils, e.g. granulocytes, to the wound site where they release cytokines and chemokines to eliminate cell debris and pathogens, and to initiate tissue repair (Peña and Martin 2024). Chemokines, such as the monocyte chemoattractant protein (MCP)-1, attract monocytes to the wound site where they develop into activated macrophages (Park and Barbul 2004). In response to pro-inflammatory mediators, such as interferon (IFN)-γ, these macrophages shift to the M1 phenotype. M1-macrophages contribute to the inflammatory phase by releasing high amounts of inflammatory cytokines, e.g. tumor necrosis factor (TNF)-α and interleukin (IL)-6 (Shapouri-Moghaddam et al. 2018).

As the inflammatory response represents the first phase in wound healing, its delayed onset and prolonged duration at an increased intensity have been recognized as key factors for impaired wound healing at elevated age (Larouche et al. 2018). Thus, the enhanced inflammatory status during wound healing in the elderly has been introduced as “inflammaging” (Pilkington et al. 2021). However, inflammaging has been determined by increased levels of inflammatory cytokines in blood samples but not in wound-tissue of human patients (Pedersen et al. 2003).

For comprehensively investigating cellular responses, molecular signaling, and extracellular matrix remodeling, rodent models provide key aspects of human wound healing (Choudhary et al. 2024). However, differences between murine and human skin repair, such as the role of specific niches of skin stem cells make it difficult to bridge the gap between preclinical and clinical studies (Zomer and Trentin 2018). Especially that a large part of murine wound closure will heal by contraction promoted by the *panniculus carnosus* muscle, is one of the most significant limitations of using loose-skinned animals to model human wounds (Masson-Meyers et al. 2020). Nevertheless, the mouse is the most commonly used model for examine wound healing, as it is easy to maintain and standardize (Zomer and Trentin 2018). Thus, inflammaging was also examined in mice and identified by increased number of M1-macrophages and T-cells in skin wound-tissue (Swift et al. 2001). Likewise, granulocytes are significantly higher in wounds of old animals (Mukai et al. 2019). However, animal studies have predominantly investigated the occurrence and distribution of immune cells in wound-tissue, without analyzing the wound milieu, i.e., the concentrations of inflammatory cytokines (Gould et al. 2015).

The transition from the inflammatory to the proliferative phase is indicated by increased levels of anti-inflammatory cytokines, which promote the monocyte development into the alternatively activated M2a-macrophages (Landen et al. 2016). High amounts of regenerative growth factors, such as hepatocyte growth factor (HGF), vascular endothelial growth factor (VEGF), and transforming growth factor (TGF)-β1 cause endothelial cells, fibroblasts and other stem cells of mesodermal origin to proliferate and secrete additional regenerative growth factors to initiate and coordinate the formation of granulation tissue and new blood vessels (Ahmad and Nawaz 2022; Almadani et al. 2021). The mesenchymal stem cells (MSCs) of the skin, particularly hair follicle stem cells as well as MSCs from the dermal adipose tissue, are crucial for the maintenance of tissue homeostasis and serve as important sources for dermal reconstruction (Langton et al. 2008; Schmidt and Horsley 2013; Sousa et al. 2024). So far, the effect of age on the release and function of the secreted growth factors has not been investigated, although they are essentially involved in granulation tissue formation, wound contraction and vascularization (Hori et al. 2012; Werner and Grose 2003; Yoshida et al. 2003).

Thus, the present study investigated the inflammatory and regenerative background of the cellular and molecular processes during skin wound healing in old female mice. Young (2 months) and old (18 months) mice received punch wounds (diameter 5 mm) on the dorsum, and the wound closure rate was monitored over 7 days. On day 1, 3 and 7 post-surgery, inflammation and regeneration were scored on the cellular level by immunohistochemical detection of granulocytes, M1-macrophages and dermal MSCs. For a molecular evaluation, the concentrations of inflammatory cytokines (MCP-1, IL-6 and TNF-α) and growth factors (VEGF, HGF, TGF-β1 and IGF-1) were determined in wound-tissue homogenates by ELISA. Since oxidative stress plays a central role in the aging process, it might also contribute to wound healing. Reactive carbonyl compounds cause “carbonyl stress”, which damages proteins (Negre-Salvayre et al. 2008). This disruption can inactivate key proteins, e.g. cytokines and growth factors (Bader and Grune 2006), with the consequence of delayed wound healing. We therefore quantified oxidized proteins in wound-tissue by the 2,4-dinitrophenylhydrazine (DNPH) method.

## Materials and methods

### Animals

WT-wild type mice (female, C57BL/6 strain) at the age of 8 weeks (n = 15; young—in telogen phase of hair growth cycle) and 18 months [n = 15; old—in telogen retention = the resting phase of hair growth cycle (Chen et al. 2014)] were purchased from Janvier Laboratories (Saint-Berthevin, Cedex, France). Because of the sex dimorphism in wound repair (Thomason et al. 2015), the present study used only female mice to examine the age effect. Since in the C57BL6/J strain, females mice heal significantly faster than male mice on days 4–5 post surgery, with a strong trend towards faster wound healing on days 1–3, we followed the recommendation to exclude sex differences when investigating wound healing (Rowland et al. 2023).

All mice were held in the facilities of the Institute of Laboratory Animal Science of the University Hospital, RWTH Aachen. Mice were kept in groups of 5 animals in plastic cages containing sawdust bedding and had access to food and water ad libitum. The mice were kept in a special barrier with a room temperature at 20–24 °C, a humidity level of 45–65% and a 12-h light/dark cycle. The animal experiments were performed according to the EU Directive 2010/63/EU for animal experiments, they followed the guidelines of the animal welfare laws and were approved by the Animal Care and Use Committee of the state of North Rhine-Westphalia, Germany (AZ-81-02.04.2021.A352).

### Anesthesia and surgery

**Fig. 1:**
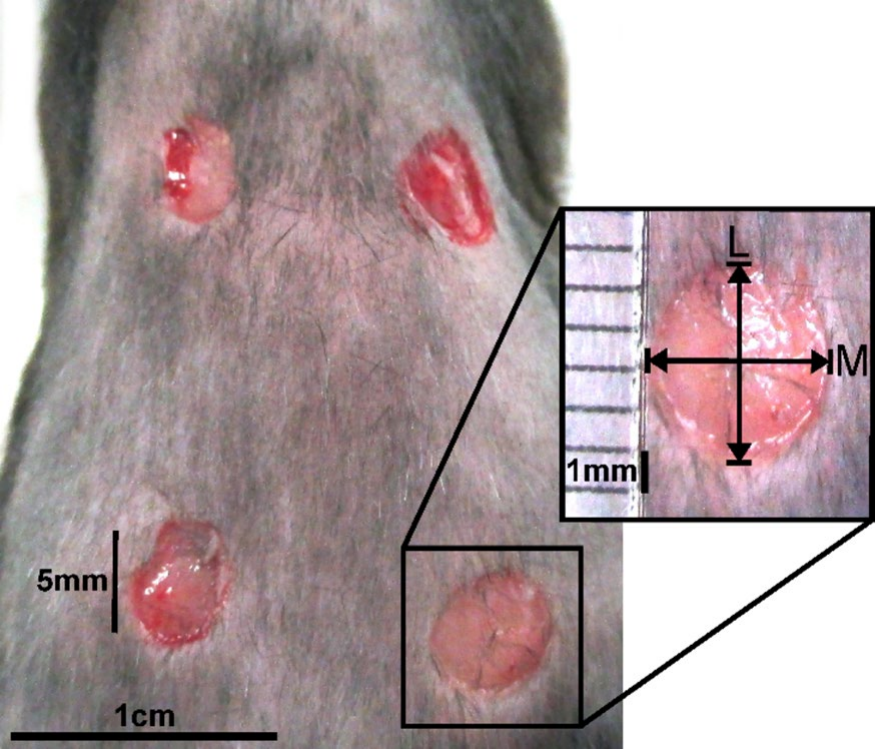
Location and dimension of excisional wounds on the dorsum of a mouse. The operation field was shaved, disinfected and the skin was wounded with a punch biopsy device, diameter 5 mm. The wound size is calculated using the larger (L) and minor (M) diameters (see magnification). Photograph was taken immediately after wounding

The general procedure of the animal experiments followed the same regime as described earlier with slight modifications (Ruhl et al. 2021b). The animals were isolated on the day prior surgery to prevent unwanted manipulation on individual wound healing, such as mutual grooming or attacks by conspecifics. The animals received metamizole for peri-operative analgesia (p.o. 125 mg/100 mL). Anesthesia was induced by 2% isoflurane inhalation, and the animals received ketamine injection for intra-operative analgesia (100 mg/kg bodyweight). All surgeries were performed under sterile conditions. To prevent the animals from hypothermia, the operating field was heated during the entire operation to 38 °C. The eyes were covered with an opaque cream (Bepanthen, Bayer, Germany), the dorsum was shaved and the skin was sterilized with Octeniderm (Schülke, Germany). With a surgical punch press (diameter: 5 mm), each mouse received four excisional wounds—proximal: 2 cm caudal to the ears; distal: 3 cm caudal to the proximal row (Fig. 1). The *panniculus carnosus* muscle was preserved to keep the function of skin contraction. Likewise, natural contraction should not be prevented by mechanical fixation of the skin by splints to prevent stress shielding that would affect the general healing process (Chen et al. 2015).

The larger (L) and minor (M) diameter of each wound (dermal border) was measured using a ruler to assess planimetric wound closure by the same person throughout the whole experiment (Fig. 1). The wound area was calculated by applying the formula: $\left(\frac{L}{2}\times \frac{M}{2}\right)\times \pi$, and the initial wound size on the day of surgery was set as 100%. The wound healing progress was determined as percentage of the wound size on day 0, respectively. After surgery, the wounds were cleaned with an Octeniderm-soaked swab. The animals were carefully returned to their home cages and monitored individually until they had fully recovered from anesthesia. Five mice per group were euthanized on day 1, 3 and 7 post-surgery by cervical dislocation. Subsequently, the diameters of the wounds were measured. All excision wounds were trimmed to 2 mm of unwounded tissue surrounding all sides of the wound in a circle. The circular piece of tissue was loosened using scissors and tweezers to peel and cut the skin from the underlying tissue. The samples were immediately frozen in liquid nitrogen and stored at − 80 °C. Three wounds (approx. 0.1 g) were homogenized in 2 mL lysis buffer (pH = 7.5, 10 mM HEPES, 0.5% Triton X-100, protease inhibitor) on ice using a tissue tearer (Thermo Fisher Scientific, Schwerte, Germany). Homogenates were centrifuged at 2000×*g* for 10 min at room temperature (RT) to remove large particles. The clear supernatant was further centrifuged at 14,000×*g* for 40 min at 4 °C, and stored at − 80 °C until used for cytokine and growth factor determination by ELISA and the protein carbonyl assay.

### Immunohistochemistry and tissue analysis

One wound-tissue sample of each mouse was fixed with 4% paraformaldehyde for 3 h, embedded in paraffin and cut into 5 µm transversal sections using a sliding microtome (pfmmedical, Cologne, Germany). Only intact sections from the center of the wound-tissue were taken for histological analysis. Heat-induced antigen retrieval was performed using a steamer (SpectraLab, Markham, Canada) with citrate buffer (pH = 6) for 30 min. The blocking was performed in two steps with Bloxall (Vector, Newark, USA) for 10 min and with 4% bovine serum albumin (BSA) for 30 min followed by washing in PBS. Sections were dried and incubated with monoclonal antibodies *vs.* Macrophage Antigen Complex-3 (Mac-3)/M1-macrophages (1:2000 μL, rat, abcam, Cambridge, United Kingdom), Lymphocyte antigen 6 complex, locus G (Ly-6G)/granulocytes (1:2000 μL, rabbit, abcam, Cambridge, United Kingdom), and Cluster of Differentiation 90 (CD90)/MSCs (1:2000 μL, rat, abcam, Cambridge, United Kingdom) diluted in blocking solution. The ImmPress HRP Horse Anti-Rabbit/Anti-Rat IgG Polymer Detection Kit (Vector, Newark, USA) containing the secondary antibody was applied following the manufacturer’s instructions. Then, the slides were washed with PBS and labelled cells were visualized using the ImmPact Amec Red Peroxidase Substrate (Vector, Newark, USA). Sections were counterstained with hematoxylin (Roth, Karlsruhe, Germany). Three regions of interest per section, along the entire wound extend, were photographed (500 × 350 µm) on an EVOS FL auto imaging system (Thermo Fisher Scientific, Waltham, USA). Of these, five randomly selected areas of equal size (50 × 50 µm) were analyzed for marked cells, which were counted manually and calculated as percentage of the total cell count using the open-source software Image J (Wayne Radband, Institutes of Health, Bethesda, USA).

### Cytokine and growth factor determination by enzyme-linked immunosorbent assay (ELISA)

To determine the concentrations of soluble factors (TNF-α, IL-6, MCP-1, HGF, VEGF, TGF-β1 and insulin-like growth factor 1 (IGF-1)), the wound extracts were thawed and analyzed by ELISA Duo-Sets (R&D Systems, Minneapolis, MN, USA) following the manufacturer’s instructions. Extinction was measured in duplicates on a microplate reader (BMG Labtech, Ortenberg, Germany).

### Determination of oxidized macromolecules concentration by protein carbonyl assay

Protein carbonyl levels were determined as an indicator of oxidized proteins in the wound homogenates collected from both the young and old mice at all three time points using the Protein Carbonyl Content Assay Kit (Merck KGaA, Darmstadt, Germany) following the manufacturers’ protocol. The results were subsequently pooled to a single data set. The protein carbonyl content was expressed as the amount (nmol) per milligram of soluble extracted protein. Whole protein concentrations of the probes were measured by DC Protein Assay (Bio-Rad Laboratories, Feldkirchen, Germany) following the instructions of the manufacturer.

### Statistics

Data of all experiments were grouped to evaluate the data for each day of testing. They were tested for normal distribution by the Shapiro–Wilk test. The results were shown as mean and standard error of the mean (± SEM). Differences between young and old animals over the course of the experiments were tested by analysis of variance followed by the Bonferroni post-hoc test; two-way ANOVA—factor 1 = age of the animals (young vs. old), factor 2 = time point of the experiment (day 1 vs. day 3 vs. day 7). Pairwise comparison between protein carbonyl content of young and old animals were analyzed using Student’s t-test. For all analyses, p-values ≤ 0.05 were considered significant.

## Results

### Wound closure rate is delayed in old mice

**Fig. 2:**
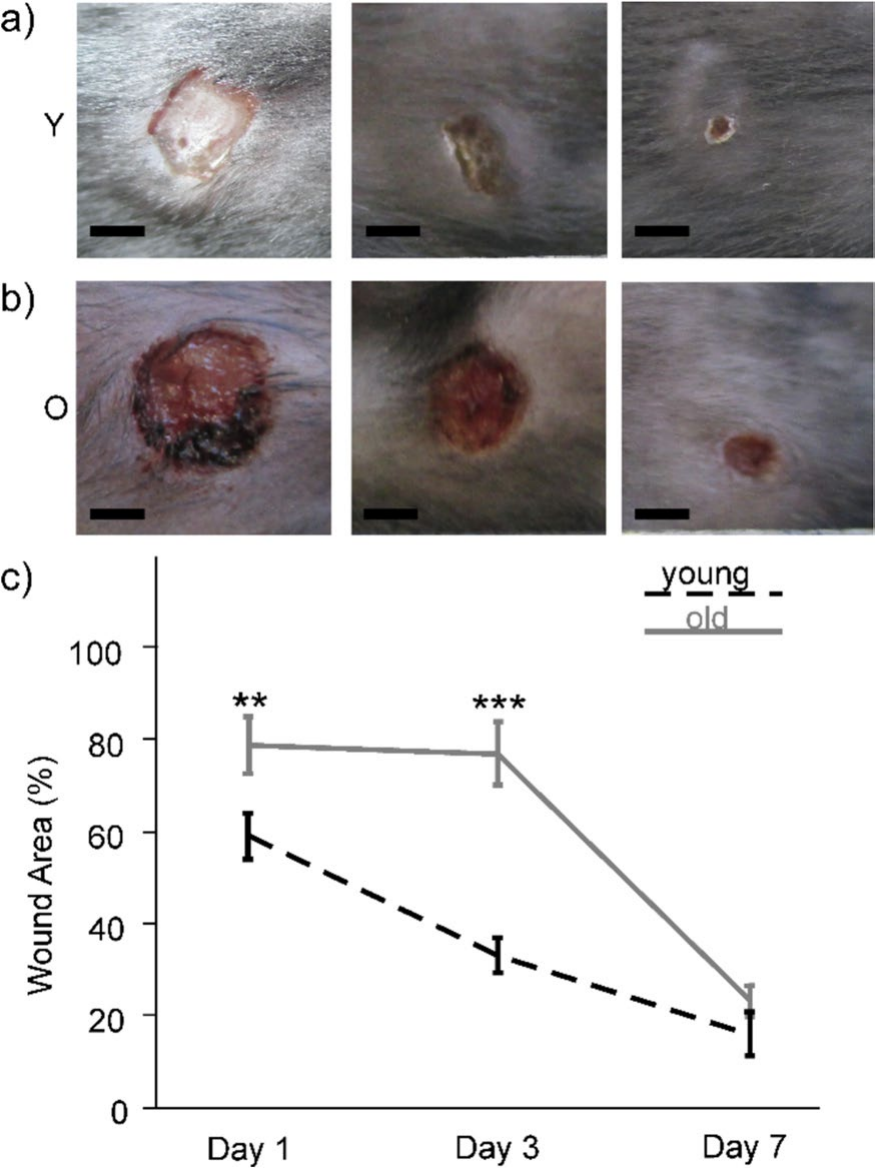
Representative images of wound closure rate in **a** young and **b** old mice on days 1, 3 and 7 post-surgery (bars = 2 mm). **c** Average of wound areas (± SEM) of excisional wounds in young (black line) and old animals (light grey line) over the course of the experiment. The number of animals was n = 5 mice for each group and time-point. Wound areas were calculated in percent to original wound size. Statistical analyses were performed by two-way ANOVA followed by Bonferroni’s post-hoc test; **p < 0.01 and ***p < 0.001 between the two age groups on the indicated time points

All mice survived during the time course of the experiments and they exhibited regular healing responses upon skin wounding, which includes weight gain and maintaining their pre-surgery behavior. The wounds were clean and free of any signs of infection or necrosis. Progressive reduction in the percentage of wound area was observed in both experimental groups (Fig. 2a, b). At day 1, wounds in old animals remained 78% (SEM ± 6%) open as compared to 58% (SEM ± 5%) in young mice (Fig. 2c). On day 3, the percentage of open wound area in the old mice was more than twice as large as in the young mice (Fig. 2c). Two-way ANOVA found significant differences for both factors, time point (F(2;111) = 45.346; p < 0.001) and age (F(1;111) = 29.675; p < 0.001), and an interaction between both factors (F(2;111) = 6.272; p = 0.003). Pairwise comparison by the Bonferroni post-hoc test found that the difference in wound closure between young and old mice was significant on day 1 (p = 0.009) as well as on day 3 (p < 0.001) post-surgery (Fig. 2c). On day 7, there was no significant difference between both groups (p = 0.357).

### Immune cell load is not increased in wounds of old mice

**Fig. 3:**
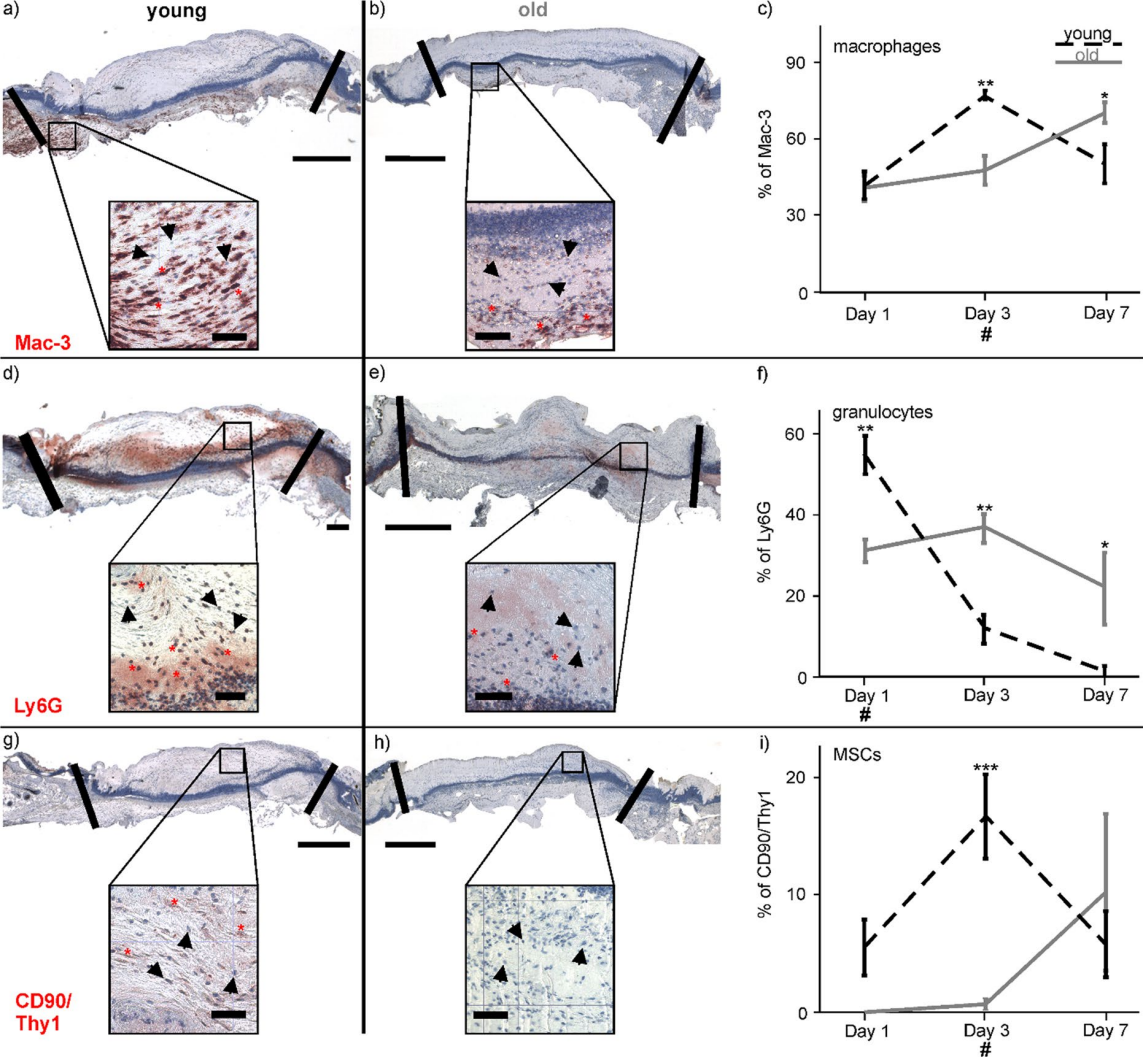
Content of Mac-3 (M1-macrophages), Ly-6G (granulocytes) and CD90/Thy1 (MSCs) in wound-tissue. Representative microphotographs of the Amec-Red stained wound sections for Mac-3 on day 3 after surgery for young (**a**) and old (**b**) mice with the percentage content of Mac-3 labelled cells in young and old mice over 7 days (**c**). Ly-6G marked cells are shown in **d, e** on day 1 post-surgery with the percentage content of Ly-6G (granulocytes) over time. **g, h** Young and old CD90/Thy1 stained cells on day 3 are shown and percentage of MSCs is presented in (**i**). The “blue band” in each photograph is a migratory tongue of epidermal cells (mainly keratinocytes) penetrating into the wound bed. Black lines in tissue overviews represent the dermal border (bars = 500 µm). Detection of labelled cells (red asterisk) *vs.* non-labelled cells (black arrow) in the magnification box (bars = 50 µm). Hash marks indicate time points of presented photographs. Statistical analyses were performed by two-way ANOVA followed by Bonferroni’s post-hoc test; *p < 0.05; **p < 0.01 and ***p < 0.001 between the groups

Immune cell and CD90^+^ MSC infiltration was evaluated on slices of wound-tissue by calculating the number of M1-macrophages (stained by Mac-3 labeling) and neutrophils (marked by Ly-6G staining) as percentage of the whole cell amount. Figure 3 shows representative microphotographs of the whole wound center of the young (Fig. 3a) and the old mice (Fig. 3b). Both age groups had equal numbers of Mac-3 positive cells on day 1 (42% ± 7%, and 41% ± 5%, respectively), while the maximum was found on day 3 for young (77% ± 2%), and on day 7 (70% ± 4%) for old mice (Fig. 3c). Two way ANOVA found significant differences for the factor time (F(2;27) = 8.314; p = 0.002), but not for the age (F(1;27) = 0.491; p = 0.490), and significant differences in the interaction of time point and age (F(2;27) = 9.265; p = 0.001). The Bonferroni post-hoc test revealed that the number of Mac-3 positive cells was significantly higher in wound-tissue of young than of old mice at day 3 (p = 0.001). At day 7, the density of Mac-3 positive cells was significantly higher in wounds of old than of young mice (p = 0.021). The peak of Mac-3 cells appeared in the young mice 4 days earlier than in the older conspecifics.

The percentage of Ly-6G positive cells in wounds of young mice was highest on day 1 (55% ± 5%) and declined afterwards, while the concentration remained constant over the course of the experimental investigation at approximately 30% in wounds of the old mice. Microphotographs of the examined wound area on day 1 showed more Ly-6G stained cells in the young (Fig. 3d) than in the old mice (Fig. 3e). The two-way ANOVA calculated significant differences between the time points (F(2;27) = 19.544, p < 0.001), no significance between the two age groups (F(1;27) = 3.024; p = 0.093), and an interaction between the time points and age (F(2;27) = 14.602; p < 0.001). Pairwise comparison using the Bonferroni post-hoc test found that numbers of Ly-6G cells were significantly higher in the wound-tissue of young mice on day 1 (p = 0.002). Higher amounts of Ly-6G marked cells were detected in tissue of the old mice on day 3 (p = 0.002) and day 7 (p = 0.011, Fig. 3f).

Marked CD90^+^ MSCs were found in wounds of the young mice on day 3 (Fig. 3g), while there were no stained cells in the tissue of the old animals (Fig. 3h). The CD90 positive cell number increased to day 3 in wounds of the young mice but not in the old mice. The peak of CD90^+^ cells in wounds of the old mice was reached on day 7. Two-way ANOVA calculated significant differences between the groups (F(1;27) = 15.540; p = 0.001), but not between the time points (F(2;17) = 2.993; p = 0.077), and a difference between time-point and age-group (F(2;17) = 3.528; p = 0.05). The following post-hoc test revealed a significantly higher percentage of CD90 positive cells in the young mice than in the old ones on day 3 (p < 0.001).

### Cytokine and growth factor concentrations

**Fig. 4:**
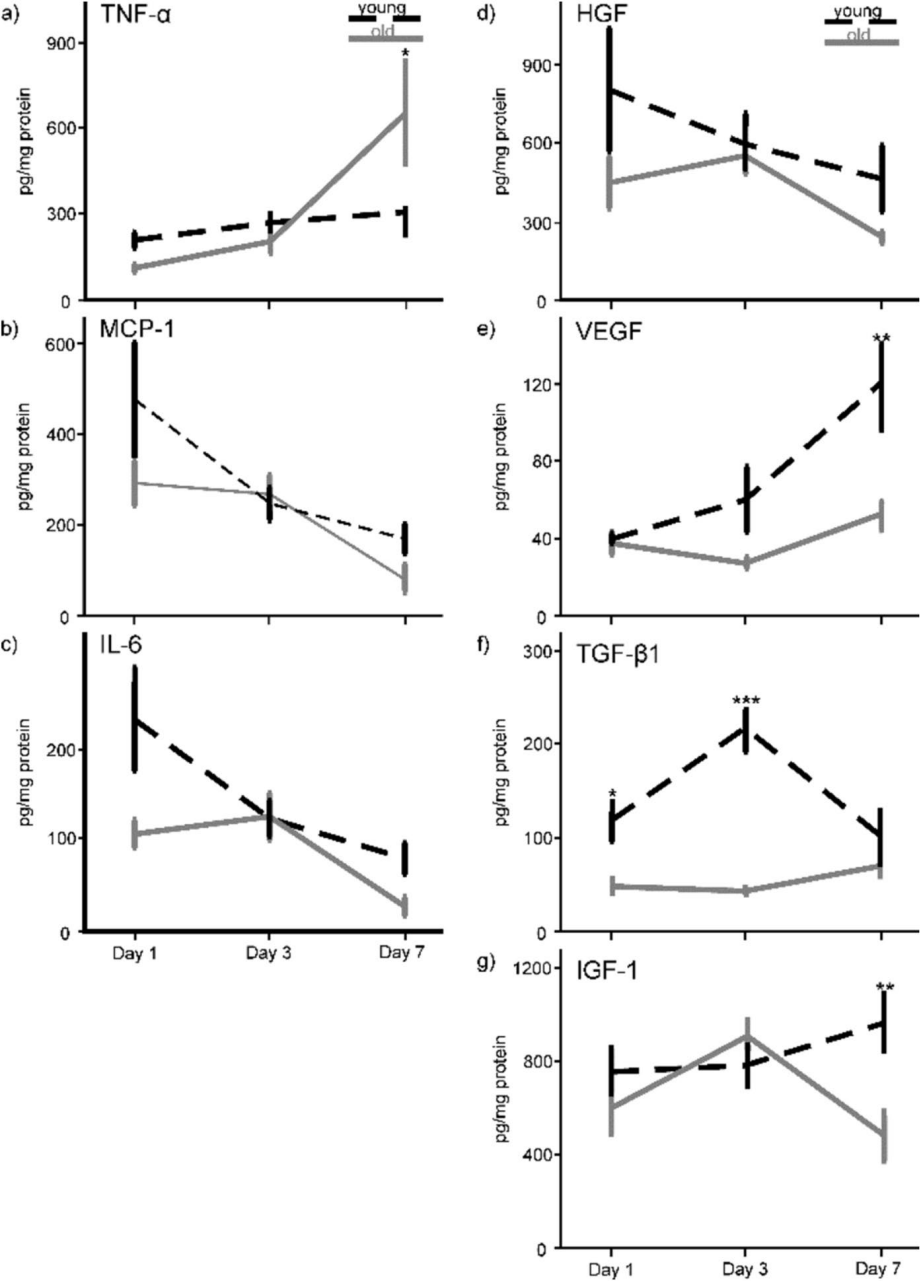
Content of pro-inflammatory cytokines: **a** TNF-α, **b** MCP-1, **c** IL-6 as well as of growth factors **d** HGF, **e** VEGF, **f** TGF-β1 and **g** IGF-1 were measured in the wound homogenates of young (black) and old (light grey) animals by enzyme-linked immunosorbent assay over the time-course of the study. The values (n = 3–5 wounds for each time-point) were normalized to the total protein content. All data are presented as means. Statistical analyses were performed by two-way ANOVA followed by Bonferroni’s post-hoc test; *p < 0.05; **p < 0.01 and ***p < 0.001 between the age groups on indicated time-points

The concentration of TNF-α in the wound-tissue increased over time in both, young and old mice (Fig. 4a). Two-way ANOVA calculated significant differences between the time points (F(2;20) = 11.143; p < 0.001), whereas not for the age groups (F(1;20) = 0.546; p = 0.468), but for the interaction between time points and age (F(2;20) = 4.187; p = 0.03). The Bonferroni post-hoc test found a significantly higher TNF-α level for wounds of the old mice than in the young ones on day 7 (p = 0.014). The pattern of MCP-1 release in old and in young mice followed a similar course, showing a maximum level at day 1 for both age groups. Two-way ANOVA found significant differences between the time points (F(2;24) = 6.626; p = 0.005), but no difference between the groups (F(1;24) = 2.110; p = 0.159) and no interaction between both factors (F(2;24) = 1.027; p = 0.373; Fig. 4b). Likewise, the amount of IL-6 indicated significant differences for the time points (F(2;22) = 8.421; p = 0.002) and the age groups (F(1;22) = 4.615; p = 0.043), but not between the time points and the age groups (F(2;22) = 1.364; p = 0.277; Fig. 4c). The maximum value of IL-6 on day 1 in the young mice was followed by a decline of the IL-6 concentration. The time course of the old animals showed a delayed maximum peak on day 3 post-surgery and a similar decrease during the remaining course of the experiment. In general, the concentration of inflammatory cytokines was higher in wound-tissue of young than of old mice, except for TNF-a which was strongly increased at day 7 of the experimental investigations. On day 3, young and old animals had similar amounts of MCP-1 and IL-6. In conclusion, wound-tissue of old animals was not characterized by an increased inflammatory level.

The two-way ANOVA revealed no significant difference in HGF levels for the age groups (F(1;21) = 2.451; p = 0.132) and the time points (F(2;21) = 1.668; p = 0.213) as well as for the interaction between the time points and the age groups (F(2;21) = 0.443; p = 0.648; Fig. 4d). The highest amount of HGF appeared on day 1 for the young mice compared to the old ones, which reached the maximum level at day 3. VEGF increased over the course of the experimental course in both age groups. Two-way ANOVA calculated significant differences for the time points (F(2;23) = 5.319; p = 0.013), the age groups (F(1;23) = 10.741; p = 0.003) and for the interaction between time and group (F(2;23) = 3.304, p = 0.05). The Bonferroni post-hoc test revealed significantly higher VEGF concentrations in wounds of the young compared to the old animals on day 7 (p = 0.001; Fig. 4e). The concentration of TGF-β1 release (Fig. 4f) in the wounds of the old mice remained at a value of around 50 pg/mg (SEM ± 4–11 pg/mg) over time. Whereas the value of the young mice was more than twice as high on day 1, reaching a value of 117 pg/mg (SEM ± 35 pg/mg) and raised to four times as high on day 3. ANOVA found significant differences for the age groups (F(1;19) = 37.697; p < 0.001) and the time points (F(2;19 = 4.388; p = 0.027), and an interaction between the age groups and the time points (F(2;19) = 8.708; p = 0.002). Pairwise comparison found significant differences in TGF-β1 concentrations between young and old mice on day 1 (p = 0.016) and day 3 (p < 0.001). ANOVA calculated no significant difference for IGF-1 between the age groups (F(1;23) = 3.426; p = 0.077) and the time points (F(2;23) = 1.142; p = 0.337), but for the interaction of time points and age groups (F(2;23) = 3.555; p = 0.045). On the last day of the examinationβ, pairwise comparison detected a significantly lower concentration of IGF-1 in the old than in the young animals (p = 0.005; Fig. 4g).

### Amount of oxidized proteins

**Fig. 5:**
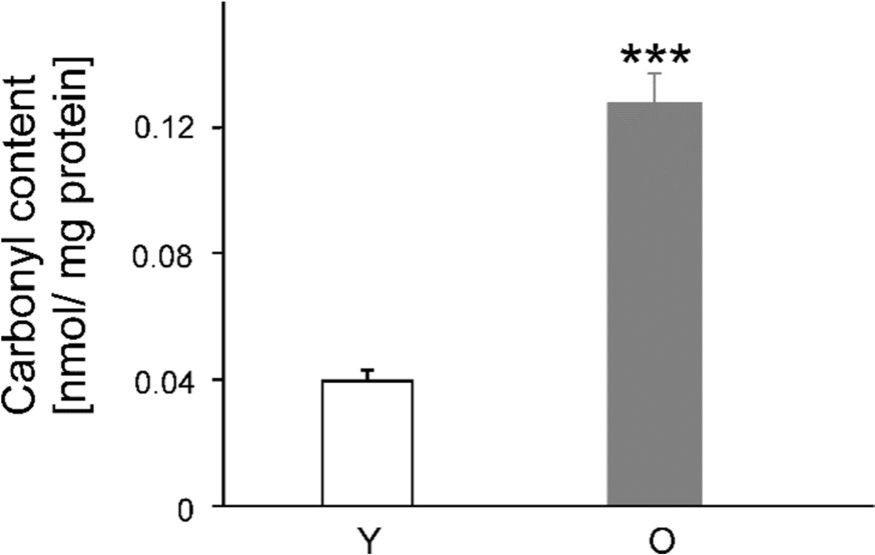
Influence of age on protein oxidation in the wound homogenates of young (Y) and old (O) mice. One wound per animal (n = 15, i.e., 5 wounds from each of the 3 time points and per group) was processed to determine protein oxidation by investigating the amount of carbonyl content calculated relative to the total protein content. The data from each time point were subsequently pooled to a single data set and are expressed as mean values + SEM. Statistical analysis was performed using the Student’s t-test for independent groups; ***p < 0.001

In the young animals, the amount of oxidized proteins in the wound-tissue homogenates from the three time points was approximately 0.04 nmol/mg protein (SEM ± 0.004 nmol/mg), while the value of the old animals was about four times higher (0.13 nmol/mg protein ± 0.009 nmol/mg). The Student’s t-test for independent variables calculated a significant difference between the two groups (t(18) = 9,046, p < 0.001; Fig. 5).

## Discussion

The necessity for an integrative approach to understand aging related effects in regenerative processes arises from the demographic shift that dramatically increases the prevalence of age-related impairments (Osareme et al. 2024). One such consequence is the reduced ability for complete skin wound repair in a regular manner (Makrantonaki et al. 2017). Comparative studies on human patients have shown that wound epithelialization is significantly delayed in elderly individuals (over 65 years of age) (Holt et al. 1992). Effective wound repair depends on factors such as a homeodynamically regulated inflammatory response followed by a physiological phase of tissue regeneration (Peña and Martin 2024). However, a prolonged duration of an intensified inflammatory reaction has been suspected to be a key factor for impaired wound healing in old organisms (Larouche et al. 2018). The immune system tends to preserve a chronic low-grade inflammatory state with increasing age, while its overall functionality and responsiveness to new infections decreases (Franceschi and Campisi 2014; Gould et al. 2015). A continuously enhanced inflammatory status, especially during impaired wound healing, has been introduced as inflammaging (Pilkington et al. 2021). The theory of inflammaging has originally arisen from studies on human patients. Elevated inflammatory cytokine levels, e.g. TNF-α and IL-6, have been measured in the blood plasma of healthy individuals, suggesting a chronically increased inflammatory state in elderly people (Pedersen et al. 2003; Salvioli et al. 2013). The same has also been found in the serum of old mice (> 12 months). It is assumed that the body enters a state of inflammaging as an adaption to immunosenescence (Pilkington et al. 2021). Immunosenescence is characterized by a declined immune system’s ability to respond effectively to new and acute challenges, such as during skin injury (Pilkington et al. 2021). Therefore, the body probably reacts to this slowed down activity with a chronically elevated inflammatory status. Contrariwise, the present study measured the acute immune response by analyzing wound-tissue instead of blood plasma, focusing on the immune system’s reaction to an acute injury rather than chronic inflammation.

In the present study, wound closure rate was delayed in old mice on days 1 and 3 post-surgery compared to young mice, which is suggestive of a delay in the inflammatory phase of wound healing. In general, this finding is in accordance with various other reports. Especially in the early observation period (up to day 6 post-wounding), 18 months old mice (Balb/cByJ; using a 3.5 mm punch device) had slower wound healing than 3 months old mice (Keylock et al. 2008). In this study, wound healing converges in both age groups on day 7, which corresponds well with our findings. The same time course of skin wound healing has been observed for C57BL/6J mice when comparing wound healing (3 mm diameter) in young (2 months) and old (20 months) animals, which differed significantly from each other up to day 4 post-surgery (Nishio et al. 2008). Reed et al. also found that the repair of dermal wounds (diameter: 6 mm) was delayed in old (24 months) versus young mice (4 months) between day 0 to day 5, but not on day 7 post-surgery (Reed et al. 2013). Similarly, the same authors found in another study impaired wound healing (6 mm diameter) in old mice (22 months; C57BL/6J) compared to young (4 months) animals until day 5 post-surgery (Reed et al. 2006). In conclusion, the results of our study are in accordance with a substantial body of evidence indicating delayed wound healing in old mice during the early inflammatory phase, with comparable outcomes across different mouse strains and wound models. We assume that the alignment of healing rates in the two age groups at day 7 was attributed to a statistical effect since we compared low numbers of animals (and wounds) of young and old mice with the consequence that the difference was still existent but not significant at the end of the observation period. In addition, a delayed catching up of the old animals’ cell populations, e.g., macrophages and MSCs, may be responsible for the convergence of wound closure rates in both age-groups by day 7 (Reed et al. 2006; Shallo et al. 2003).

The infiltration of immune and connective tissue cells into the wound is essential for orchestrating the inflammatory response, followed by tissue regeneration and the extracellular matrix formation. Thus, it is crucial for the proper progression of the whole wound healing process (Rodrigues et al. 2019; Wilkinson and Hardman 2020). In our study, we observed a delayed granulocyte peak in the old mice at day 3. However, the granulocyte numbers of the old animals were permanently high, which could be interpreted as a chronically inflammatory status. The number of M1-macrophages increased constantly until day 7 in the old mice meaning a delayed and prolonged inflammatory response, while the numbers of M1-macrophages peaked at day 3 and declined afterwards in the young mice. Nevertheless, M1-macrophage and granulocyte numbers did not differ between old and young mice, however, there was a time-shift in the occurrences of both cell populations between the two age groups. The existing literature on age-related changes in immune cell migration is inconsistent. Mukai et al. created 4 mm skin wounds on mice and found that the granulocyte number was significantly higher in old than in young mice on day 7 post-surgery (Mukai et al. 2019). Conversely, no difference was found between the age-groups for numbers of M1-macrophages. On the other hand, Swift et al. observed no change in neutrophil but in M1-macrophage infiltration in excisional wounds of old mice (Swift et al. 2001). These wounds contained 56% more M1-macrophages than wounds from young animals between day 1 and 5 post-surgery. This result is also comparable to a study on human wound-tissue, which found an increase in the number of M1-macrophages in wounds of old patients (Ashcroft et al. 1998). Contrasting studies observed a delayed, but reduced immune cell number after dermal injury in old mice (Brubaker et al. 2013). However, other studies report an unchanged immune cell count and delayed cell-infiltration (Nishio et al. 2008). For instance, these findings are very similar to ours as we also failed to find a difference in the immune cell load but a delay for the cell-infiltration. Therein, the neutrophil infiltration peaked early at 6 h post-surgery in young mice, while it was delayed until 24 h after wounding in old mice. The peak of M1-macrophage numbers appeared at 48 h in both age groups, but declined earlier in young mice. Our findings showed a delayed immune cell migration and an associated shift of the inflammatory phase, which could have contributed to the delayed wound healing in the old mice until day 3. A comparable result has also been found by another study reporting that the numbers of M1-macrophages and B lymphocytes peaked at day 3 after wounding in 6 months young animals, whereas it is delayed and smaller in 30 months old C57BL/lcrfa’ mice (Ashcroft et al. 1997). It has been hypothesized that old mice have an impaired inflammatory response with persistence of neutrophils in wounds because an advanced age could be associated with impaired neutrophil chemotaxis (Brubaker et al. 2013). A delay in the macrophage occurrence and/or activity could be an explanation for the delay of the whole wound healing process in the early stages since these cells exert multiple beneficial effects on wound repair. In the early stages (day 1), M1-macrophages remove cell debris and pathogens, while later during the repair process, starting from day 3, M2a-macrophages promote tissue repair by secreting growth factors (Aitcheson et al. 2021). Another explanation for a delay in immune cell response may be an age-related decline in the quantity of chemoattractant cytokines released into the wound-tissue. Nevertheless, we did not observe differences in the chemoattractant cytokine MCP-1 in our study.

While increased inflammation in old organisms has been predominantly determined by cell infiltration, their secretory activity, *i.e.* the release of inflammatory cytokines, has been hardly investigated. Swift et al. measured MCP-1 levels in wound homogenates during an observation period of 7 days (Swift et al. 2001). They detected higher MCP-1 amounts in the wounds of old mice exclusively during the early hours post-injury, but not on the days afterwards, which corresponds well with our findings. Comparable results were also observed in a burn injury model. MCP-1 concentrations were lower in wound-tissue of old (18 months) than of young (3 months) mice one day after injury (Shallo et al. 2003). At day 4 post-surgery, both age groups reached similar levels of MCP-1, which is consistent with the results of the present study. Therefore, we suggest that inflammatory reactions are not necessarily increased in old age, which challenges the prevailing inflammaging-hypothesis.

During physiological skin wound healing, the inflammatory stage merges with the proliferative phase, which is orchestrated by the interaction of immune cells, MSCs, fibroblasts, keratinocytes, and endothelial cells, amongst others. In general, a higher density and activity of dermal MSCs is associated with faster healing and reduced scar formation (Diaz-Garcia et al. 2021; Yang et al. 2020). However, the presence and physiology of dermal MSCs in skin wounds is affected by age. The activity of the hair follicle MSCs orchestrate the hair growth cycle, i.e., growth (anagen), apoptosis-driven regression (catagen) and relative quiescence (telogen) (Lin et al. 2022). These cells contribute to accelerated wound closure when mice are wounded in the late anagen compared with the catagen or telogen hair-cycle stage (Ansell et al. 2011). However, the activity of hair follicle MSCs is triggered as a response to propagating signals or activators from their macro-environment, which is heavily impaired by the age of the animal (Chen et al. 2014). Furthermore, Amini-Nik et al. (2022) used a thermal injury model to investigate burn wound healing in mice. They found a deficiency of CD90 positive MSCs in the wounds of old (52–54 weeks) *vs.* young (8–12 weeks) animals, which has been attributed to a decrease in cell migration from the adjacent intact tissue. It is therefore possible that we failed to detect MSCs in wounds of the old mice because these cells resided in their biological niche, e.g. the hair follicle, and they were, thus, not participating in the dermal regeneration.

There have been only a few murine studies on wound healing that have measured the concentrations of regenerative growth factors in wound-tissue. Using a standardized excisional injury model, lower levels of VEGF and fibroblast growth factor-2 were determined in wound-tissue of old compared to young mice (Swift et al. 1999), which complies well with the results of the present study. Further evidence indicates that in old mice, both basal and wound-induced expression of various fibroblast growth factors are reduced, e.g. FGF11 (Komi-Kuramochi et al. 2005). The authors interpret this finding at least partly responsible for the age-related slower healing of wounds. VEGF and IGF-1 are known for their proangiogenic activity, and it is suggested that a decline in angiogenic growth factor concentrations may account for delayed neovascularization during tissue regeneration in old mice (Aghdam et al. 2012; Swift et al. 1999). Furthermore, TGF-β1 is a potent chemokine for leukocytes and fibroblasts in wound healing and it also mediates tissue debridement by modulating the activity of M1-macrophages (Xia et al. 2016). This helps to protect the surrounding healthy tissue and prepares the wound for granulation tissue formation (Barrientos et al. 2008). Thus, impaired growth factor release may also hinder the timely migration and activity pattern of cells at the wound site, which could further impair the healing process (Wang et al. 2006). Our findings on the concentration of soluble factors in the wound milieu of young and old mice, e.g. inflammatory cytokines and growth factors, suggest that differences in the regenerative capacity rather than inflammatory scores might account for age-related differences in wound healing.

Another explanation for the delayed response of immune cells and MSCs in our study could be that oxidative damage reduced the effectiveness of key proteins, such as cytokines and growth factors. The regenerative capacity of the skin declines with age in association with increased accumulation of senescent cells (Thanapaul et al. 2021). Cells can enter a status of senescence, if they undergo oxidative damage without having adequate anti-oxidative protective mechanisms, e.g. as a result of the natural aging process (Davalli et al. 2016). Reactive carbonyl compounds induce the ‘carbonyl stress’ characterized by the formation of adducts and cross-links via disulfide bonds on proteins, which progressively leads to impaired protein function and an altered protein structure (Negre-Salvayre et al. 2008). Such oxidative modifications have been extensively studied both in vivo and in vitro (Bader and Grune 2006; Merker et al. 2001; Stadtman 1992)**.** In addition, the degradation of oxidized macromolecules is only possible to a limited extend, leading to the accumulation of dysfunctional proteins in aged cells (Jung et al. 2007; Sitte et al. 2000). In our study, we detected increased protein carbonyl content in the wounds of old mice, suggesting that a number of proteins were oxidized, which can result in a change of their properties. A recent study investigated the effects of oxidation on the human epidermal growth factor (hEGF) protein (Yusupov et al. 2018). Oxidation shows a significant effect on the structural conformation and the binding affinity with its receptor (EGFR), and this will most probably cause inhibition of the cell growth or proliferation. Moreover, Mazière et al. demonstrate that an overproduction of ROS leads to inactivation of the EGF signalling pathway (Mazière et al. 2003) and that oxidation is able to disrupt the insulin signalling pathway (Mazière et al. 2004). In concrete terms, this would mean that the growth factors in our study were not only secreted to a lesser extent but they were also less effective in old mice. This suggests that the overall effect of these growth factors has been reduced to an even greater extent than would be expected from the reduction in secretion alone. A similar phenomenon may also apply to the pro-inflammatory cytokines. Korn et al. (2001) showed that oxidative stress factors like hydrogen peroxide inhibit the activity of TNF-α, by inactivating its kinase complex. According to our measurements, the cytokines were actually on similar levels. However, considering that the concentration of oxidized proteins was higher in the old than in the young mice, their overall activity could have been impaired. In order to prove those conclusions, future research could focus on identifying specific oxidized proteins and assessing the impact of their functional impairment.

## Conclusion

The present study demonstrates a novel and important role of inflammation and regeneration during wound-tissue repair in old mice. Wound healing of 2 months old mice was characterized by elevated concentrations of the growth factors VEGF, TGF-ß1 and IGF-1 and an earlier infiltration of MSCs, accelerating the healing process by encouraging the formation of new cells and tissues. Contrariwise, wound healing in old animals was significantly delayed one and three days after wounding. As a new aspect, we observed no differences in the amount of proinflammatory cytokines and a delayed but not increased immune cell infiltration in the wound-tissue of old animals, which could potentially overturn existing theories. Our findings suggest that inflammation might not be the key factor for delayed wound healing in old mice, but we found alternative age-related-alterations in the regenerative potential that could explain a slower healing with age. Based on our findings, we propose a therapeutic strategy in regenerative medicine of aging which could recover the age-related impairment of the endogen regenerative processes. For example, the stimulation of the endocannabinoid system appears to be a promising option, as cannabinoids supress the release of inflammatory cytokines by immune cells, increase the pro-regenerative potential of adult subcutaneous stem cells, and also possess antioxidant properties (Dawidowicz et al. 2021; Ruhl et al. 2021a, c).

## Data Availability

The datasets analyzed during the current study are available from the corresponding author on reasonable request.

## Abbreviations

CD90: Cluster of Differentiation 90
EGFR: Epidermal growth factor receptor
hEGF: Human epidermal growth factor
HGF: Hepatocyte growth factor
IGF: Insulin-like growth factor
IL-6: Interleukin-6
KC: Keratinocyte-derived chemokine
Ly-6G: Lymphocyte antigen 6 complex, locus G
MAC-3: Macrophage antigen complex-3
MCP-1: Monocyte chemoattractant protein-1
MIP-2: Macrophage inflammatory protein-2
MSC: Mesenchymal stem cell
RT: Room temperature
TGF-β1: Transforming growth factor Beta1
TNF-α: Tumor necrosis factor Alpha
VEGF: Vascular endothelial growth factor
